# Tick Tock—A Matter of Time: Two Cases of Babesia Acquired in Urban Newark, NJ

**DOI:** 10.1155/2024/3912571

**Published:** 2024-11-29

**Authors:** Jorge A. Caceda, Afshan Iqbal, Kristy Bono, Diana Finkel, Eli Goshorn

**Affiliations:** ^1^Rutgers New Jersey Medical School, 185 S. Orange Avenue, Newark 07103, New Jersey, USA; ^2^Department of Medicine, Division of Infectious Disease, Rutgers, The State University of New Jersey, 185 South Orange Ave Medical Science Building I-689, Newark 07103, New Jersey, USA

**Keywords:** Babesiosis, epidemiology, parasite, urban

## Abstract

Babesiosis is a parasitic tick-borne infectious disease that is well elucidated in medical literature and known to be endemic to the Midwest and northeast United States. However, like other infectious diseases, its epidemiology is subject to change. This case report documents two cases with clinical presentations that deviate from what is expected in typical cases of Babesiosis. Two patients presented to a safety-net hospital in Newark, NJ, during the summer of 2022 with nonspecific symptoms. The first patient had a history of polysubstance use disorder and presented with bilateral leg pain, drowsiness, exertional dyspnea, back pain, and chest pain. The second patient had recently returned from a trip to Guatemala and presented with subjective fevers, generalized myalgias, malaise, headaches, and chills. Both patients underwent similar workups yielding a diagnosis of Babesiosis. Of note, neither patient had recently spent time in wooded areas. Ultimately, both patients were treated for Babesiosis with resolution of their presenting symptoms. These two cases suggest that the epidemiology of Babesiosis is changing and provide a clinical workflow for diagnosing and managing this disease in a modern healthcare setting.

## 1. Introduction

Babesiosis is a malaria-like illness caused by *Babesia microti* which is endemic to the upper mid-western and northeast region of the United States [1]. It is transmitted by the nymphal stage of the *Ixodes scapularis* tick, more commonly known as the “deer tick” [1, 2]. The disease occurs mostly during the warmer season, particularly between the months of May to September [1]. The primary reservoir is thought to be the white-footed mouse but other smaller mammals, such as shrews, may also play a role [1]. For this reason, cases of Babesiosis tend to center around populations in wooded areas and areas with a prominent deer population [2]. However, as with any other infectious disease, its epidemiology is subject to change based on outside forces such as climate change. Prior spatial environmental models have predicted the expansion of tick-borne illnesses into greater geographic latitudes such as Canada, and away from regions like the southeastern United States [3].

Following a tick bite, the incubation period varies, but typically ranges from one to 4 weeks [1]. Symptoms can include, fever, fatigue, malaise, and myalgias, as well as complications, such as hemolytic anemia, jaundice, and acute kidney injury [1, 2]. Another documented category of feared complications of Babesiosis includes splenic pathologies such as infarct, rupture, and splenomegaly [4, 5]. Additionally, there have been reports of Babesiosis being transmitted via blood transfusion, further amplifying this disease's clinical significance [6]. Cumulatively, Babesiosis is an infectious disease that remains clinically relevant and has been showing signs of epidemiologic changes in the last few decades.

## 2. Patient Information

### 2.1. Patient 1

A 63-year-old man with a past medical history of hypertension and polysubstance use disorder (alcohol, tobacco, intranasal heroin) presented to the emergency department (ED) in July 2022 with two weeks of bilateral leg pain. He also complained of drowsiness, exertional dyspnea, back pain, and chest pain. He denied fevers, chills, night sweats, weight loss, shortness of breath, cough, prolonged immobilization, and recent travel. For substance use, he reported taking three shots of alcohol daily, insufflating 14-15 bags of heroin every other day, and smoking one pack of cigarettes over the course of a week. He denied injection drug use. He was employed as a cleaner, but also took on various odd jobs. He resided in urban Newark, NJ, and denied exposure to any wooded areas or travel out of the state for the last 10 years.

In the ED, his vital signs showed a blood pressure of 129/63, heart rate of 60, temperature of 98.2 F (36.8°C), and oxygen saturation of 98% on room air. Physical exam revealed bilateral pitting edema. He underwent CT angiography of the chest that was negative for pulmonary embolism. High-sensitivity troponin resulted negative. ECG demonstrated sinus bradycardia with a ventricular rate of 56 beats per minute. ED providers assessed him to be low risk for a major adverse cardiac event. He was subsequently discharged home with instructions to follow up with his primary care physician.

Ten days later, returned to the ED with persistent symptoms. He denied any new exposures but recalled being bitten by an unknown insect in his backyard 1–2 weeks prior. Vital signs showed a blood pressure of 122/64, heart rate of 78, temperature of 98.2 F (36.8°C), and oxygen saturation of 98% on room air. Laboratory testing demonstrated anemia, leukopenia and thrombocytopenia, which was not present at the previous visit (Table 1). Blood smears were performed, demonstrating a parasitemia index of 1.9%. *Babesia* serology and PCR, *Ehrlichia* PCR, *Anaplasma* PCR, Lyme serologies, and HIV screen were also ordered. The patient was empirically treated with oral atovaquone 750 mg every 12 h and oral azithromycin 500 mg daily and admitted. The patient was normotensive as an inpatient and was not taking any antihypertensive as an outpatient, so no antihypertensive therapy was given.

His symptoms rapidly improved in the days following admission and fevers resolved. Formal pathology review of the patient's blood smear revealed intracellular parasites consistent with Babesiosis. Serial blood smears were conducted to trend the parasitemia (Table 2). Pathogen-specific work up revealed elevated *Babesia microti* IgG and positive *Babesia* PCR, and positive for Lyme IgG and IgM (Table 3). The patient was discharged on hospital day five with instructions to continue atovaquone 750 mg every 12 h and azithromycin 500 mg daily to complete a 10-day total course of therapy. After discharge, he was prescribed a 14-day course of oral doxycycline 100 mg every 12 h for treatment of early Lyme disease. The patient was provided an outpatient appointment in the infectious disease clinic; however, he was lost to follow up.

### 2.2. Patient 2

A 67-year-old man with a past medical history of Type II diabetes presented to the ED in late August 2022 with a 3-day history of subjective fever that worsened in the evenings. He also complained of generalized myalgias, malaise, headaches, and chills. Approximately 3 weeks ago, he returned from a 2-week vacation in a rural area of Guatemala. He denied hiking or camping, but did experience many mosquito bites during his trip. He did not recall any animal exposures, insect or tick bites after returning. Review of systems was negative for bleeding, bruising, or rash. He did not take any malaria prophylaxis prior to or during his travels and had not previously received a diagnosis of malaria. He was employed as a superintendent at an apartment complex and was involved in the necessary repairs and maintenance.

Upon presentation to the ED, vitals included a blood pressure of 116/67, heart rate of 60, temperature of 98 (36.7), and oxygen saturation of 100% on room air. Lab results were significant for elevated transaminase, bilirubin, and alkaline phosphatase, along with mild anemia and thrombocytopenia (Table 4). There was an initial concern for malaria due to recent travel to Guatemala. Malaria smear returned positive with a parasitemia index of 2.7%. A rapid malaria antigen test (binaxNOW) was negative. However, due to the relatively low sensitivity of this test for *Plasmodium ovale* [7], the patient was started on empiric therapy for malaria. With oral artemether-lumefantrine, 4 tablets every 8 h for the first 2 doses and then 4 tablets every 12 h for the next 4 doses for a total of a 3-day course. Additionally, the patient's pre-existing Type II diabetes was medically managed with sliding scale insulin and his glimepiride and metformin were held. *Babesia* serology and PCR were obtained while a final pathologist read of the blood smears was in progress.

**Figure 1:**
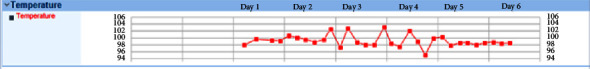
Temperature during admission. Trend of Patient #2's temperature throughout admission.

The patient experienced subjective improvement in his symptoms on hospital day one. However, he developed persistent high fevers during his inpatient stay (Figure 1). Due to ongoing fevers despite antimalarial therapy, empiric therapy for Babesiosis was initiated with oral atovaquone 750 mg every 12 h and oral azithromycin 500 mg daily on hospital day three. Additionally, antipyretic therapy was given with oral acetaminophen 650 mg every 6 h as needed. Lyme serology and PCR for *Anaplasma* and *Ehrlichia* were also ordered. Empiric oral doxycycline 100 mg every 12 h was added on day four while Lyme and Babesiosis testing was in process.

**Figure 2:**
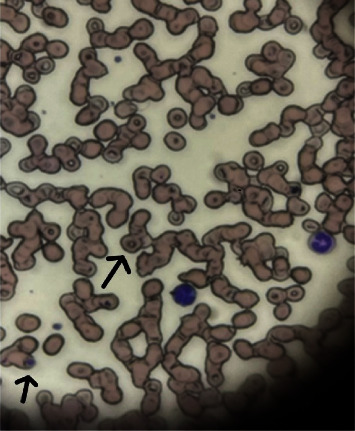
Blood smear from patient #2 showing parasitic forms consistent with Babesia. Ring forms, rare extracellular rings, and intracellular chromatin dots are present-two examples of which are demonstrated with arrows.

The pathology read of the blood smears demonstrated parasitic forms consistent with Babesiosis. Ring forms, rare extracellular rings, and intracellular chromatin dots were also present (Figure 2). The patient's parasitemia levels decreased throughout admission (Table 5). The patient was discharged home on day six, with instructions to complete a 10-day course of oral atovaquone 750 mg every 12 h and azithromycin 500 mg daily. Testing for Lyme and rickettsial infection was pending at the time of discharge and he continued on oral doxycycline 100 mg every 12 h until results were available. He was seen in the infectious disease clinic 10-day after hospital discharge. By this time, pathogen-specific testing results were available, significant for only *Babesia microti* DNA (Table 6). Doxycycline was discontinued and he completed the prescribed course of oral atovaquone and azithromycin. He felt well and was discharged with counseling on measures to avoid tick exposure.

## 3. Discussion

Babesiosis is an infectious malaria-like disease that is primarily transmitted by tick bites in the northeastern and mid-western states of the United States [1, 2]. In this article, we report two cases of Babesiosis in city-dwelling patients who lacked the classical environmental exposures for this disease.

In general, tick-borne diseases have increased in the United States, from 40,795 reported in 2011 to 50,856 in 2019, roughly an increase of 25% [8]. A study by the CDC assessing trends of Babesiosis between 2011 and 2019 showed increased annual incidence of the disease in Connecticut, Maine, New Hampshire, Massachusetts, New Jersey, New York, Rhode Island, and Vermont (*p* < 0.001) [9]. The largest percent changes in incidence were reported in Connecticut, Vermont, Maine and New Hampshire; however, the largest number of cases was in New York [9]. Thus, major increases were seen in the northeast United States.

Additional states bordering those mentioned above may also be at risk, as increased case counts and infection rates with *Babesia* have been reported in areas which previously had absent or very low infection rates. These include the Canadian provinces of Manitoba and Ontario, as well as Delaware, Ohio, Illinois, the Dakotas, Virginia, and West Virginia [10, 11]. There are several studies based in different European countries, which show that more densely-populated areas such as urban and suburban counties are now seeing increases in rates of Babesiosis and other tick-borne diseases [12–14]. In the United States, there is evidence of increasing amounts of Babesiosis in urban and suburban settings as well. New Jersey, the most densely populated state in the United States, has demonstrated steady increases in Babesiosis over the last few years [15]. These findings were also seen in Essex country, where the reported cases were diagnosed [15].

Climate change is one possible cause of the increase in Babesiosis. Generally, this disease occurs during times of warm temperature, allowing the larvae to hatch [1]. Consistently rising temperatures might be contributing to an increase in favorable conditions for *Babesia* [3]. Even daily temperature changes have been reported to affect the risk of disease transmission [16]. The changing epidemiology of Babesiosis necessitates a change in thinking on the part of the medical community. Other tick-borne diseases can present similarly, and coinfection with multiple tick-borne diseases is common. Further cases of Babesiosis in city inhabitants should be documented to elucidate any patterns in clinical presentation and patient outcomes.

Another factor that may be contributing to the increase in Babesiosis includes population dynamics. For example, aging populations may have less immunocompetent individuals and therefore have greater incidence of symptomatic Babesiosis [17]. The overall immune status of the population can determine the severity of the infection and thus the amount of patients that interact with healthcare. Based on the immune system of the host, Babesiosis clinical presentation can range from asymptomatic all the way to life-threatening [18]. Furthermore, those with asplenia are more likely to develop Babesiosis and severe infection [4]. *Babesia* spp. specifically target host erythrocytes. The parasite invades erythrocytes and appears in a ring form [1]. It can continually replicate to form its characteristic “maltese cross”, after maturation; it can be released into the bloodstream to affect more erythrocytes [1]. People without spleens are unable to clear infected erythrocytes, and thus are more susceptible [4]. All of these factors can be further compounded by the overall increase in population density, especially in urban areas which can facilitate the spread of disease.

*Babesia* can cause a severe illness on its own, as documented in these cases, but it does not exclusively exist in isolation. In fact, coinfection with other tick-borne microbes such as *Borrelia burgdorferi* has been well documented. Interestingly, some studies have shown worse outcomes with Lyme coinfection, while others have opposed those findings. In 1996, Krause et al. performed a community-based annual serosurvey and clinical cohort study reporting that the amount of symptoms and duration of illness in patients with concomitant Lyme disease and Babesiosis were greater than in patients with either infection alone [19]. In another study, *Babesia* coinfection with Lyme disease or anaplasmosis reduced the risk for hospitalization (RR = 0.73, CI-0.53-0.99, *p*=0.03) [20]. One explanation for this inconsistency is that when infection with one organism alone or coinfection is superimposed on other comorbidities, it makes it more difficult to draw consistent conclusions as to whether or not coinfection is inherently more clinically deleterious.

The differential diagnosis for acute-onset fever, thrombocytopenia, and elevated liver enzymes is broad and spans multiple etiologies. Infectious causes for this presentation include Lyme disease, Babesiosis, anaplasmosis, ehrlichiosis, leptospirosis, and viral hepatitis. Noninfectious causes of this clinical presentation can also widely vary, but a common differential in this patient population includes alcoholic hepatitis and drug-induced liver injury. The cases presented here indicate that physicians should consider Babesiosis in any patient presenting with a malaria-like illness who resides in or has recently spent time in the northeastern US, even if they have not spent time in wooded areas; failure to do so may lead to a delayed or missed diagnosis.

## Figures and Tables

**Table 1:** Patient 1 laboratory data.

	Index ER visit	2nd ER visit
WBC (range 4.0–11.0)	4.4 × 10^∗^3/μL	3.8 × 10^∗^3/μL
Hemoglobin (range 14.0–18.0)	11.3 g/dL	9.6 g/dL
Platelets (range 150–450)	173 × 10^∗^3/μL	78 × 10^∗^3/μL
MCV	81.9 fL	79.6 fL
LDH	Not performed	413 μ/L
Reticulocytes	Not performed	1.9%
ALT	16 μ/L	23 μ/L
AST	20 μ/L	32 μ/L
CPK	Not performed	

*Note:* Basic laboratory blood data from Patient #1 showing the development of anemia, leukopenia, and thrombocytopenia during his second visit to the emergency department.

Abbreviation: WBC, white blood cell count.

**Table 2:** Parasitemia from blood smears.

	Day 1 (%)	Day 1 (7 h later) (%)	Day 3 (%)	Day 4 (%)
Parasite index	1.9	1.7	1.0	0.5

*Note:* Regular parasite smear results showing downtrend of parasitemia in Patient #1.

**Table 3:** Parasite and HIV work up.

*Babesia microti* IgM (negative is < 1:10)	<1:10
*Babesia microti* IgG (negative is < 1:10)	1:320
*Babesia* PCR	DNA detected
Lyme IgG EIA	Positive
Lyme IgM EIA	Positive
Human granulocytic Ehrlich IgG	Negative
Human granulocytic Ehrlich IgM	Negative
HIV 4th generation test	Nonreactive

*Note:* Parasite and HIV work up for Patient #1 significant for elevated *Babesia microti* IgG. Positive *Babesia* PCR, and positive screen for Lyme IgG and IgM.

Abbreviations: EIA, enzyme immunoassay; HIV, human immunodeficiency virus; IgG, immunoglobulin G; IgM, immunoglobulin M; PCR, polymerase chain reaction; WBC, white blood cell count.

**Table 4:** Patient 2 laboratory data.

WBC (range 4.0–11.0)	10.2 × 10^∗^3/μL
Hemoglobin (range 14.0–18.0)	13.0 × 10^∗^3/μL
Platelets (range 150–450)	42 × 10^∗^3/μL
AST (range 0–40)	79 U/L
ALT (range 0–41)	90 U/L
Alkaline phosphatase (40–130)	150 U/L
Total bilirubin (range <1 mg/dL)	1.4 mg/dL
Direct bilirubin (range <0.3 mg/dL)	0.5 mg/dL

*Note:* Basic laboratory blood data from Patient #2 showing elevated liver function tests, anemia, and thrombocytopenia.

Abbreviations: ALT, alanine transaminase; AST, aspartate aminotransferase; WBC, white blood cell count.

**Table 5:** Parasitemia index from blood smears.

	Day 1 (%)	Day 2 (%)	Day 3 (*Babesia* treatment starts) (%)	Day 4 (%)	Day 5 (%)	Day 6
Parasite index	2.7	2.5	2.4	1.2	Present, < 0.1	Negative

*Note:* Regular parasite smear results showing resolution of parasitemia in Patient #2.

**Table 6:** Parasite work up.

*Babesia microti* PCR	DNA detected
*Anaplasma* PCR	Not detected
*Ehrlichia chaffeensis* PCR	Not detected
Lyme total antibody EIA	Negative
*Plasmodium* species PCR	Not detected

*Note:* Parasite work up from Patient #2 showing elevated *Babesia microti* DNA.

Abbreviations: EIA, enzyme immunoassay; PCR, polymerase chain reaction.

## Data Availability

The supporting data used to support the findings of this study are included within the article.
